# Differential Modulation of Serum Albumin and Butyrylcholinesterase Following Pemafibrate Treatment in MASLD: A Post Hoc Analysis of the PEMA-FL Trial

**DOI:** 10.3390/metabo16100750

**Published:** 2026-10-06

**Authors:** Ryohei Tanigawa, Rohit Loomba, Yuichiro Eguchi, Hirokazu Takahashi, Hideki Suganami, Andrey Belous, Atsushi Nakajima

**Affiliations:** 1 Global Clinical Development Department, Kowa Company Ltd., 4-14, 3-Chome, Nihonbashi-Honcho Chuo-ku, Tokyo 103-8433, Japan; 2 Department of Hepatology, Division of Metabolism, Faculty of Medicine, Saga University, 1-1, 5-Chome, Nabeshima, Saga 849-8501, Japan; 3 MASLD Research Center, Division of Gastroenterology and Hepatology, Department of Medicine, University of California San Diego, La Jolla, CA 92037, USA; 4 Loco Medical General Institute, 1-1178, Mikazukichokanada, Ogi 845-0032, Japan; 5 Clinical Data Science Department, Kowa Company Ltd., 4-14, 3-Chome, Nihonbashi-Honcho Chuo-ku, Tokyo 103-8433, Japan; 6 Clinical Development, Kowa Pharmaceutical Development, Inc., 500 Boylston Street, 17th Floor, Boston, MA 02116, USA; abelous@kowa.com; 7 Department of Gastroenterology, International University of Health and Welfare Atami Hospital, 13-1 Higashikaigancho, Atami 413-0012, Japan

**Keywords:** pemafibrate, MASLD, PPARα, albumin, butyrylcholinesterase, liver-derived circulating biomarkers

## Abstract

**Highlights:**

**What are the main findings?**

- In this exploratory post hoc analysis of patients with non-cirrhotic MASLD, pemafibrate treatment was associated with a sustained increase in serum albumin and a sustained decrease in serum butyrylcholinesterase compared with placebo.
- Serum albumin and butyrylcholinesterase showed divergent longitudinal responses during pemafibrate treatment, although both are liver-derived circulating biomarkers.

**What are the implications of the main findings?**

- Changes in serum albumin and butyrylcholinesterase during pemafibrate treatment should be interpreted individually and should not be considered direct evidence of changes in overall hepatic synthetic function.
- The mechanisms and clinical significance of these divergent biomarker responses remain uncertain and warrant further mechanistic and prospective investigation.

**Abstract:**

**Background:** Pemafibrate, a selective peroxisome proliferator-activated receptor-α (PPARα) modulator, improves liver stiffness and biochemistry in metabolic dysfunction-associated steatotic liver disease (MASLD). The longitudinal responses of serum albumin and butyrylcholinesterase during pemafibrate treatment remain incompletely characterized. We performed an exploratory post hoc analysis to characterize the effects of pemafibrate on these biomarkers in patients with non-cirrhotic MASLD. **Methods:** In this exploratory post hoc analysis of the randomized PEMA-FL trial, 118 patients with non-cirrhotic MASLD received pemafibrate (0.4 mg/day) or placebo for 72 weeks. Longitudinal changes in serum albumin and butyrylcholinesterase were evaluated. Between-group differences were estimated using ANCOVA, with MMRM performed as a sensitivity analysis. **Results:** Serum albumin and butyrylcholinesterase remained largely unchanged in the placebo group. In contrast, treatment was associated with a sustained increase in serum albumin and a sustained decrease in serum butyrylcholinesterase from week 4 through week 72. At week 72, the adjusted between-group difference in serum albumin was 0.18 g/dL (95% confidence interval [CI], 0.11 to 0.24). The adjusted between-group difference in serum butyrylcholinesterase was −33.5 U/L (95% CI, −45.2 to −21.7). Findings from the MMRM sensitivity analysis were consistent with those of the primary ANCOVA analyses. **Conclusions**: In this exploratory post hoc analysis, pemafibrate treatment was associated with a sustained increase in serum albumin and a sustained decrease in butyrylcholinesterase activity in patients with non-cirrhotic MASLD. These biomarkers should be interpreted individually because the mechanisms and clinical significance of their differing responses remain uncertain. ClinicalTrials.gov: NCT03350165; registered on 20 November 2017.

## 1. Introduction

Metabolic dysfunction-associated steatotic liver disease (MASLD) has traditionally been regarded as a disorder primarily characterized by hepatic lipid accumulation [1]. Nevertheless, abnormalities in hepatic protein metabolism are increasingly recognized as clinically important during disease progression, particularly because serum albumin independently predicts liver-related outcomes in patients with MASLD [2]. Serum albumin and butyrylcholinesterase are two commonly used liver-derived circulating biomarkers in routine clinical practice [3,4], and they are often assumed to reflect related aspects of liver physiology. However, whether these two proteins in fact respond uniformly to a therapeutic intervention has not been directly tested.

As liver fibrosis advances, alterations in liver-related metabolic and nutritional status become increasingly evident and are frequently accompanied by malnutrition and sarcopenia, both of which are independently associated with poorer quality of life, hepatic decompensation, and mortality [5,6]. Therefore, biomarkers reflecting these aspects of liver-related metabolic and nutritional status may provide clinically relevant information beyond conventional liver biochemistry.

Pemafibrate is a selective peroxisome proliferator-activated receptor-α (PPARα) modulator (SPPARMα) developed for the treatment of dyslipidemia [7,8,9,10,11,12]. Beyond its lipid-modifying effects, selective PPARα activation enhances fatty acid oxidation and regulates multiple metabolic pathways involved in energy homeostasis [12,13,14]. In the randomized, double-blind, placebo-controlled PEMA-FL trial, pemafibrate significantly improved liver stiffness and liver biochemistry despite the absence of a significant reduction in hepatic fat content [15]. Subsequent post hoc analyses demonstrated that pemafibrate has an ameliorating effect on portal hypertension-related parameters, including platelet count and spleen volume [16]. Notably, favorable changes in markers of liver-muscle metabolism were also observed, including increased circulating carnitine concentrations and reduced plasma myostatin levels, suggesting improved liver-muscle metabolic homeostasis [17].

Whether these metabolic effects extend to liver-derived circulating biomarkers such as serum albumin and butyrylcholinesterase—and specifically, whether albumin and butyrylcholinesterase are affected in a concordant or divergent manner—remains unknown. Serum albumin is an established prognostic biomarker in MASLD, with lower concentrations independently associated with hepatic decompensation, hepatocellular carcinoma, and mortality [2]. Although both proteins are synthesized predominantly by hepatocytes, serum albumin is influenced by multiple factors, including nutritional status, systemic inflammation, and protein turnover, whereas butyrylcholinesterase may be influenced by hepatic steatosis and insulin resistance [4,18]. These distinct biological determinants raise the possibility that the biomarkers may exhibit different longitudinal responses during a metabolic intervention such as selective PPARα activation.

We therefore performed an exploratory post hoc analysis of the randomized PEMA-FL trial to characterize the longitudinal responses of serum albumin and butyrylcholinesterase during pemafibrate treatment in patients with non-cirrhotic MASLD.

## 2. Materials and Methods

### 2.1. Study Design

This was a post hoc analysis of the randomized, double-blind, placebo-controlled PEMA-FL trial (ClinicalTrials.gov: NCT03350165; registered on 20 November 2017), the design and primary results of which have been reported previously [15]. Briefly, 118 patients with non-cirrhotic metabolic dysfunction-associated steatotic liver disease (MASLD) were randomized to receive pemafibrate (0.2 mg twice daily) or placebo for 72 weeks. The present exploratory post hoc analysis evaluated the longitudinal responses of serum albumin and butyrylcholinesterase following selective PPARα activation. The present study adhered to the principles of the Declaration of Helsinki, Good Clinical Practice Guidelines, and applicable local regulations. Institutional review board approval was obtained from each participating institution (e.g., the institutional review board of Yokohama City University Hospital; approval number: 17-363), and all participants provided written informed consent.

The participants were randomized 1:1 to receive pemafibrate (0.2 mg twice daily, total 0.4 mg/day) or a matching placebo for a 72-week treatment period with no change in dietary and/or exercise guidance throughout the screening and treatment period.

Random allocation was performed using an interactive web response system (CAC Croit Corporation, Tokyo, Japan; currently EPS Corporation due to corporate merger). The key inclusion criteria were magnetic resonance imaging-proton density fat fraction (MRI-PDFF)-confirmed steatosis, increased liver stiffness according to magnetic resonance elastography (MRE) findings, and persistently elevated alanine aminotransferase (ALT) levels. The exclusion criteria included excessive alcohol intake, extreme body mass index (BMI), poorly controlled diabetes, renal impairment, biliary obstruction, liver cirrhosis, and liver diseases other than MASLD. The full criteria are summarized in Supplementary Table S1 and Supplementary Figure S1.

### 2.2. Laboratory Assessments

Serum albumin and butyrylcholinesterase (pseudocholinesterase) were measured centrally by LSI Medience Corporation (Tokyo, Japan) according to the PEMA-FL protocol.

Serum butyrylcholinesterase was reported in U/L. Additional methodological details regarding the butyrylcholinesterase assay, including the analytical method and reference range, are provided in the Supplementary Table S2.

### 2.3. Statistical Analysis

Baseline values for albumin and butyrylcholinesterase were defined as the mean of the screening and week-0 measurements. Continuous outcomes were analyzed using analysis of covariance, with treatment group as a fixed effect and the corresponding baseline value as a covariate. Between-group least-squares mean differences with 95% confidence intervals were estimated at each scheduled visit. For the visit-specific ANCOVA analyses, missing observations were not imputed, and all available observations at each visit were analyzed. No multiplicity adjustment was applied because all analyses were exploratory. Therefore, all reported *p* values should be interpreted as nominal.

As a sensitivity analysis, a mixed model for repeated measures (MMRM) was performed. The MMRM included baseline value as a covariate and treatment group, visit, and treatment-by-visit interaction as fixed effects. An unstructured covariance matrix was used to model within-subject correlations among repeated measurements over time. Model parameters were estimated using restricted maximum likelihood, and degrees of freedom were calculated using the Kenward-Roger approximation. The MMRM analysis was conducted under the missing-at-random (MAR) assumption and estimated the treatment effect that would have been observed if measurements had remained observable throughout follow-up, conditional on the observed data included in the model. The MMRM analysis was performed using SAS version 9.4 (SAS Institute Inc., Cary, NC, USA).

Exploratory correlation analyses were performed to assess the relationship between serum albumin concentration and butyrylcholinesterase activity at baseline and the relationship between their changes from baseline at week 72. Pearson correlation coefficients and 90% density ellipses were calculated separately by treatment group. The correlation analyses were descriptive, and differences between treatment-group-specific correlation coefficients were not formally tested. JMP software version (17.0.0; JMP Statistical Discovery LLC, Cary, NC, USA) was used to perform the correlation analyses, including calculation of Pearson correlation coefficients (r) and 90% density ellipses.

## 3. Results

### 3.1. Characteristics of the Study Population

Between December 2017 and October 2018, 180 individuals were screened, and 118 were randomized to the placebo group (*n* = 60) or the pemafibrate group (*n* = 58). A total of 112 participants completed the 72-week treatment period (placebo, *n* = 59; pemafibrate, *n* = 53). Six participants (placebo, *n* = 1; pemafibrate, *n* = 5) discontinued treatment due to adverse events or withdrawal. All 118 randomized participants were included in the full analysis set used for this exploratory post hoc analysis. Baseline demographic and disease characteristics were generally balanced between the treatment groups (Table 1). The mean age of the study population was 53 years; 36.4% of participants had type 2 diabetes mellitus, 57.6% had hyperlipidemia, and 66.9% met the criteria for metabolic syndrome. The mean baseline hepatic fat content measured by MRI-PDFF was 18.4%, and the mean liver stiffness measured by MRE was 3.1 kPa. More than half of the participants had a fibrosis stage of F2 or higher.

### 3.2. Differential Effects of Pemafibrate on Serum Albumin and Butyrylcholinesterase

**Figure 1:**
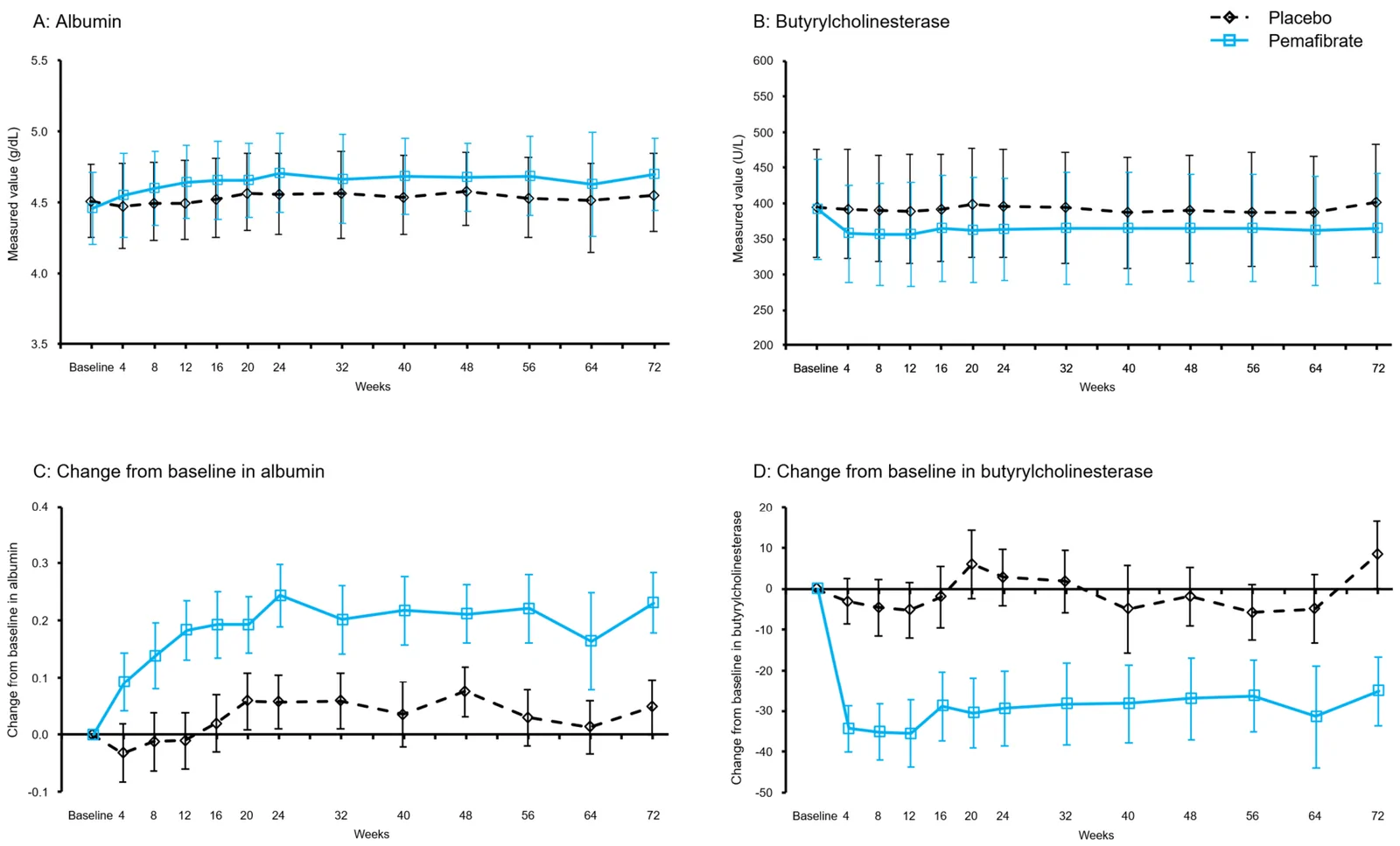
Longitudinal changes in serum albumin and butyrylcholinesterase during pemafibrate treatment. (**A**) Observed serum albumin concentrations, (**B**) observed serum butyrylcholinesterase activities, (**C**) baseline-adjusted changes in serum albumin, and (**D**) baseline-adjusted changes in serum butyrylcholinesterase over 72 weeks in the placebo and pemafibrate groups. Observed values are presented as means with standard deviations. Changes from baseline are presented as least-squares means estimated using analysis of covariance, with the corresponding baseline value included as a covariate. Error bars for changes from baseline represent 95% confidence intervals.

Serum albumin and butyrylcholinesterase exhibited distinct and divergent longitudinal responses to pemafibrate treatment (Table 2, Figure 1).

In the pemafibrate group, serum albumin increased from week 4, the first post-baseline assessment, and remained elevated throughout the 72-week treatment period. At week 72, the least-squares mean between-group difference in the change from baseline was 0.18 g/dL (95% confidence interval [CI], 0.11 to 0.24). Serum albumin remained largely unchanged in the placebo group.

In contrast, serum butyrylcholinesterase decreased from week 4 after the initiation of pemafibrate treatment and remained lower throughout the 72-week treatment period, whereas little change was observed in the placebo group. At week 72, the least-squares mean between-group difference in the change from baseline was −33.5 U/L (95% CI, −45.2 to −21.7). Serum butyrylcholinesterase also remained largely unchanged in the placebo group.

### 3.3. Relationship Between Changes in Serum Albumin and Butyrylcholinesterase

**Figure 2:**
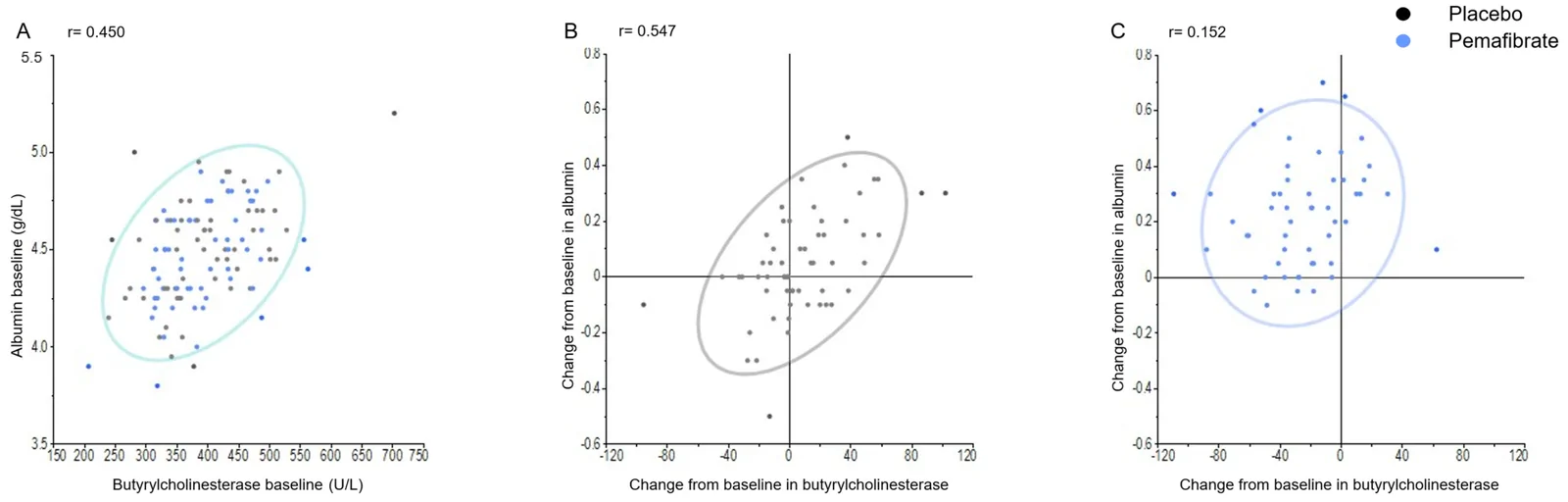
Exploratory relationships between serum albumin and butyrylcholinesterase. (**A**) Relationship between serum albumin concentration and serum butyrylcholinesterase activity at baseline in the placebo and pemafibrate groups. Relationship between the change from baseline in serum albumin concentration and the change from baseline in serum butyrylcholinesterase activity at week 72 in the (**B**) placebo and (**C**) pemafibrate groups. Black dots indicate placebo-treated participants, and blue dots indicate pemafibrate-treated participants. Ellipses represent 90% density ellipses. Pearson correlation coefficients are shown for descriptive purposes. Differences between treatment-group-specific correlation coefficients were not formally tested.

Baseline albumin was moderately correlated with baseline butyrylcholinesterase (r = 0.450). The distributions of participants were similar between the placebo and pemafibrate groups across the observed range of baseline albumin and butyrylcholinesterase values (Figure 2A). At week 72, the Pearson correlation coefficient between the changes from baseline in serum albumin and butyrylcholinesterase activity was 0.547 in the placebo group and 0.152 in the pemafibrate group (Figure 2B,C). These correlation patterns were evaluated descriptively, and the difference between the correlation coefficients was not formally tested.

### 3.4. MMRM Sensitivity Analysis

To assess the robustness of the visit-specific ANCOVA analyses, an MMRM sensitivity analysis was performed. The MMRM results were generally consistent with those obtained from the visit-specific ANCOVA analyses, demonstrating a sustained increase in serum albumin and a sustained decrease in serum butyrylcholinesterase during pemafibrate treatment compared with placebo (Supplementary Table S3 and Supplementary Figure S2).

## 4. Discussion

The present study showed sustained but divergent longitudinal responses of serum albumin and butyrylcholinesterase during pemafibrate treatment in patients with non-cirrhotic MASLD: albumin increased whereas butyrylcholinesterase decreased throughout treatment. Although both are liver-derived circulating biomarkers, the observed responses should not be interpreted as direct evidence of changes in overall hepatic synthetic function or hepatic protein metabolism. Rather, the findings indicate that serum albumin and butyrylcholinesterase should be interpreted individually during pemafibrate treatment. In particular, a decline in butyrylcholinesterase should not automatically be interpreted as deterioration of hepatic synthetic function.

Serum albumin is also influenced by systemic inflammation, nutritional status, plasma volume, vascular permeability, and protein turnover [2,3]. In contrast, a recent deeply phenotyped human study demonstrated that higher plasma butyrylcholinesterase activity was independently associated with greater liver fat content and lower insulin sensitivity, while reductions in butyrylcholinesterase during two lifestyle interventions tracked reductions in liver fat and improvements in insulin sensitivity [18]. Notably, comparable associations with glycemia or insulin sensitivity were not observed for albumin in that study, suggesting that butyrylcholinesterase and albumin may be influenced by different biological and metabolic determinants. Although hepatic fat content assessed by MRI-PDFF did not significantly decrease with pemafibrate in the parent PEMA-FL trial, treatment was associated with significant improvements in ALT, gamma-glutamyl transferase (GGT), and alkaline phosphatase (ALP) [15]. Accordingly, the observed decline in butyrylcholinesterase should not necessarily be interpreted as evidence of worsening hepatic synthetic function. Given the previously reported associations of butyrylcholinesterase with hepatic steatosis and insulin resistance [18], its reduction during pemafibrate treatment may have potential metabolic relevance. Nevertheless, the biological significance of this finding remains uncertain.

Exploratory correlation analyses were performed to evaluate the relationship between serum albumin and butyrylcholinesterase. At week 72, the change in albumin was moderately correlated with the change in butyrylcholinesterase in the placebo group, whereas the corresponding correlation coefficient was smaller in the pemafibrate group. Together with the divergent longitudinal responses, these findings suggest that serum albumin and butyrylcholinesterase did not show parallel patterns during pemafibrate treatment. The biological basis of these observations remains uncertain.

Selective PPARα activation regulates multiple metabolic pathways through transcriptional regulation of fatty acid transport, mitochondrial β-oxidation, ketogenesis, lipoprotein metabolism, and energy substrate utilization [7,8,9,10,11,12,13,14]. Together with previous PEMA-FL analyses showing improvements in liver stiffness [15], portal hypertension-related parameters such as platelet count and spleen volume [16], and favorable changes in liver-muscle metabolic markers—specifically increased circulating carnitine and reduced plasma myostatin [17], the present findings add to the range of biochemical and metabolic changes observed during pemafibrate treatment. Although these findings expand the range of biochemical and metabolic changes observed during pemafibrate treatment, the present study was not designed to investigate the mechanisms underlying the observed changes in serum albumin and butyrylcholinesterase. Therefore, the biological basis of these biomarker responses remains uncertain and requires further investigation.

From a clinical perspective, the sustained increase in serum albumin deserves further investigation, although its clinical implications should be interpreted with appropriate caution. Serum albumin is an established prognostic biomarker across chronic liver diseases, with lower concentrations associated with hepatic decompensation, hepatocellular carcinoma, and mortality [2]. In the present study, albumin rose early after treatment initiation, and the adjusted between-group difference in the change from baseline was 0.18 g/dL (95% CI, 0.11 to 0.24) at week 72. However, the clinical significance of an increase of this magnitude in patients with non-cirrhotic MASLD remains uncertain. The present study did not evaluate hepatic decompensation, hepatocellular carcinoma, mortality, or other long-term clinical outcomes, and no prognostic or survival benefit can be inferred from the observed albumin change. Whether pemafibrate produces a clinically meaningful albumin response and whether changes in serum albumin during treatment are associated with subsequent clinical outcomes in patients with more advanced fibrosis or cirrhosis remains to be tested prospectively.

In a recently reported hypertriglyceridemia cohort, a similar sustained increase in serum albumin was observed during pemafibrate treatment in patients with concomitant primary biliary cholangitis (PBC) [19], a cholestatic liver disease with a pathophysiology distinct from MASLD. Although this observation suggests that an increase in serum albumin during pemafibrate treatment may not be restricted to MASLD, the two studies differed in their populations, underlying liver diseases, clinical settings, and study designs. Therefore, the consistency and clinical relevance of the albumin response across different chronic liver diseases require confirmation in prospective studies.

### Limitations

This study has several limitations. First, this was an exploratory post hoc analysis of a randomized clinical trial and was not originally designed to evaluate serum albumin or butyrylcholinesterase as primary outcomes. In addition, the sample size was relatively small, and participant attrition occurred during the 72-week follow-up period.

Second, no adjustment for multiplicity was performed; therefore, all reported *p* values should be interpreted as nominal, and the findings should be regarded as exploratory.

Third, the study did not directly assess protein synthesis, protein degradation, amino acid flux, plasma volume, nutritional status, systemic inflammation, insulin sensitivity, or mechanisms regulating butyrylcholinesterase. Consequently, the observed biomarker changes cannot be interpreted as direct evidence of changes in hepatic synthetic function, hepatic protein metabolism, or metabolic improvement.

Fourth, the correlation analyses were exploratory and descriptive. Differences between correlation coefficients in the placebo and pemafibrate groups were not formally tested, and the observed correlation patterns do not establish that pemafibrate altered the underlying biological relationship between serum albumin and butyrylcholinesterase.

Fifth, cardiovascular disease was not a formal exclusion criterion in the parent PEMA-FL trial, and detailed information on cardiovascular disease and concomitant medications was not evaluated in the present post hoc analysis. In addition, multiple post hoc analyses have previously been reported from the same parent trial. These factors should be considered when interpreting the present findings.

Sixth, the clinical significance of the observed increase in serum albumin remains uncertain. Because clinical outcomes were not evaluated, no inference can be made regarding liver-related events, hepatic decompensation, or survival.

Finally, the study included only patients with non-cirrhotic MASLD, which limits the generalizability of the findings to patients with advanced fibrosis, cirrhosis, or other chronic liver diseases.

## 5. Conclusions

Pemafibrate treatment was associated with sustained but divergent longitudinal responses of serum albumin and butyrylcholinesterase in patients with non-cirrhotic MASLD. These findings indicate that serum albumin and butyrylcholinesterase, although both liver-derived circulating biomarkers, may not necessarily exhibit parallel responses during pemafibrate treatment and should therefore be interpreted individually. The mechanisms underlying these biomarker responses remain uncertain, and the observed changes should not be interpreted as direct evidence of changes in hepatic synthetic function or hepatic protein metabolism. Further mechanistic and prospective studies are needed to clarify the biological basis and clinical significance of these observations.

## Figures and Tables

**Table 1:** Baseline characteristics of the study population.

	Placebo (*n* = 60)	Pemafibrate (*n* = 58)
Demographics		
Age (years)	53.3 (16.6)	53.2 (12.5)
Male	37 (61.7)	31 (53.4)
Comorbidities		
Type 2 diabetes ^a^	25 (41.7)	18 (31.0)
Hyperlipidemia ^a^	37 (61.7)	31 (53.4)
Hypertension ^a^	28 (46.7)	26 (44.8)
MASH		
No	1 (1.7)	3 (5.2)
Yes	17 (28.3)	19 (32.8)
Unknown	42 (70.0)	36 (62.1)
Metabolic syndrome ^b^	41 (68.3)	38 (65.5)
Metabolic factors		
Body weight (kg)	82.0 (24.8)	80.0 (16.8)
Body mass index (kg/m^2^)	29.8 (6.5)	29.6 (4.9)
Waist circumference (cm)	99.9 (15.1)	100.2 (10.4)
Liver image		
Liver fat content by MRI-PDFF (%)	18.05 (5.48)	18.75 (6.89)
MRE-based liver stiffness (kPa)	3.02 (0.44)	3.24 (0.81)
MRE-based LSM stage		
≤1	30 (50.0)	26 (44.8)
2	25 (41.7)	21 (36.2)
≥3	5 (8.3)	11 (19.0)
Clinical laboratory tests		
ALT (U/L)	94.6 (49.4)	82.8 (36.6)
AST (U/L)	57.3 (26.5)	54.2 (20.7)
GGT (U/L)	78.0 (54.1)	85.3 (73.4)
ALP (U/L)	254 (74)	260 (76)
Albumin (g/dL)	4.51 (0.26)	4.46 (0.25)
Butyrylcholinesterase (U/L)	394.2 (82.2)	392.0 (70.1)
Total bilirubin (mg/dL)	0.98 (0.41)	0.94 (0.40)
Platelets (10^10^/L)	23.1 (5.85)	23.0 (5.81)

Data are expressed as mean (standard deviation) or *n* (%). ^a^: Based on physician assessment and diagnosis. ^b^: Defined as increased waist circumference plus ≥ 2 risk factors (abnormal serum lipids, blood pressure, or fasting plasma glucose) per Japanese metabolic syndrome criteria. ALP: alkaline phosphatase; ALT: alanine aminotransferase; AST: aspartate aminotransferase; GGT: gamma-glutamyl transferase; MASH: metabolic dysfunction-associated steatohepatitis; MRE: magnetic resonance elastography; MRI: magnetic resonance imaging; PDFF: proton density fat fraction.

**Table 2:** Results of albumin and butyrylcholinesterase.

	Baseline	Week 4	Week 12	Week 24	Week 48	Week 72
Albumin (g/dL)						
Placebo						
*n*	60	60	60	59	59	59
Measured value	4.51 (0.26)	4.47 (0.30)	4.50 (0.30)	4.56 (0.29)	4.58 (0.27)	4.55 (0.29)
Change	-	−0.03 [−0.08 to 0.02]	−0.01 [−0.06 to 0.04]	0.06 [0.01 to 0.10]	0.08 [0.03 to 0.12]	0.05 [0.00 to 0.10]
*p* value	-	0.208	0.660	0.019	0.001	0.050
Pemafibrate						
*n*	58	58	58	57	55	53
Measured value	4.46 (0.25)	4.55 (0.29)	4.64 (0.26)	4.71 (0.28)	4.68 (0.24)	4.70 (0.26)
Change	-	0.09 [0.04 to 0.14]	0.18 [0.13 to 0.24]	0.24 [0.19 to 0.30]	0.21 [0.16 to 0.26]	0.23 [0.18 to 0.28]
*p* value	-	<0.001	<0.001	<0.001	<0.001	<0.001
LS mean difference						
Estimate	-	0.12 [0.05 to 0.19]	0.19 [0.12 to 0.25]	0.18 [0.11 to 0.25]	0.13 [0.07 to 0.19]	0.18 [0.11 to 0.24]
*p* value	-	0.001	<0.001	<0.001	<0.001	<0.001
Butyrylcholinesterase (U/L)						
Placebo						
*n*	60	60	60	59	59	59
Measured value	394.2 (82.2)	391.2 (84.8)	389.0 (79.6)	395.4 (80.4)	390.8 (77.1)	400.9 (81.9)
Change	-	−3.1 [−8.6 to 2.5]	−5.2 [−12.0 to 1.6]	2.8 [−4.1 to 9.7]	−1.8 [−9.0 to 5.3]	8.3 [0.0 to 16.6]
*p* value	-	0.274	0.129	0.423	0.610	0.049
Pemafibrate						
*n*	58	58	58	57	55	53
Measured value	392.0 (70.1)	357.7 (68.2)	356.4 (73.1)	363.9 (71.3)	365.6 (75.6)	365 (77.0)
Change	-	−34.3 [−40.0 to −28.7]	−35.6 [−43.8 to −27.4]	−29.4 [−38.5 to −20.2]	−27.0 [−37.1 to −16.9]	−25.1 [−33.6 to −16.6]
*p* value	-	<0.001	<0.001	<0.001	<0.001	<0.001
LS mean difference						
Estimate	-	−31.3 [−39.2 to −23.5]	−30.5 [−40.9 to −20.2]	−32.1 [−43.2 to −21.0]	−25.2 [−37.0 to −13.3]	−33.5 [−45.2 to −21.7]
*p* value	-	<0.001	<0.001	<0.001	<0.001	<0.001

Data are expressed as observed means (standard deviations) or least-squares means (95% confidence intervals) for changes from baseline. No adjustment for multiplicity was performed; therefore, all *p* values should be interpreted as nominal. LS, least-squares.

## Data Availability

The data presented in this study are available on request from the corresponding author. The data are not publicly available due to patient privacy considerations and contractual restrictions related to the clinical trial data.

## Supplementary Materials

The following supporting information can be downloaded at: https://www.mdpi.com/article/10.3390/metabo16100750/s1, Figure S1: Study design of the PEMA-FL Study (NCT03350165); Figure S2: Changes from baseline in serum albumin and butyrylcholinesterase in the MMRM sensitivity analysis; Table S1: Inclusion and exclusion criteria; Table S2: Methodological details of serum butyrylcholinesterase measurements; Table S3: MMRM results for changes from baseline in serum albumin and butyrylcholinesterase.

## Abbreviations

The following abbreviations are used in this manuscript:

ALP	Alkaline phosphatase
ALT	Alanine aminotransferase
ANCOVA	Analysis of covariance
BMI	Body mass index
CI	Confidence interval
GGT	Gamma-glutamyl transferase
MASH	Metabolic dysfunction-associated steatohepatitis
MASLD	Metabolic dysfunction-associated steatotic liver disease
MRE	Magnetic resonance elastography
MRI	Magnetic resonance imaging
MMRM	Mixed model for repeated measures
PDFF	Proton density fat fraction
PPAR	Peroxisome proliferator-activated receptor-α
SPPARM	Selective peroxisome proliferator-activated receptor-α modulator
