# Supplementary material for: Differential Modulation of Serum Albumin and Butyrylcholinesterase Following Pemafibrate Treatment in MASLD: A Post Hoc Analysis of the PEMA-FL Trial
Source: Metabolites. 2026 Oct 6;16(10):750. doi: 10.3390/metabo16100750 (PMC13706844; doi:10.3390/metabo16100750)
Supplement: Supplementary file 1 [file metabolites-16-00750-s001.zip › Supplementary Materials.pdf]

Figure S1: Study design of the PEMA-FL Study (NCT03350165).

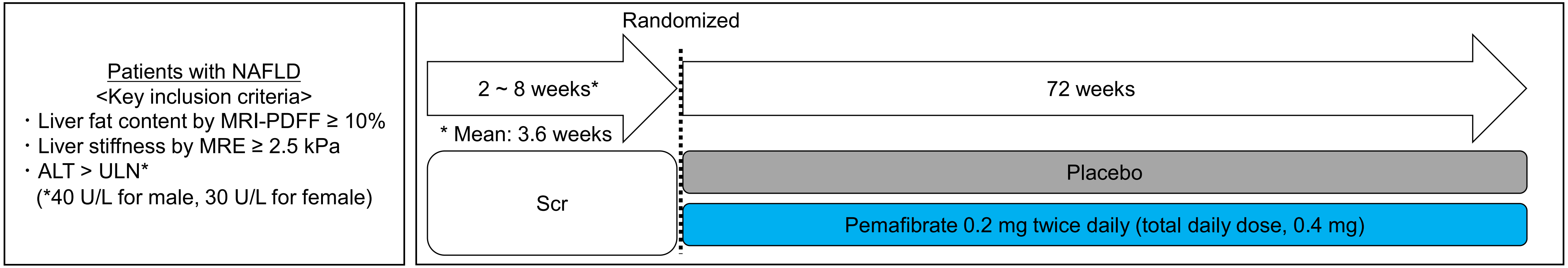


| | Screening period | Treatment period | | | | | | | | | | | | |
|---|---|---|---|---|---|---|---|---|---|---|---|---|---|---|
| Visit (week) | −8 ~ −2 | 0 | 4 | 8 | 12 | 16 | 20 | 24 | 32 | 40 | 48 | 56 | 64 | 72 |
| Liver imaging (MRI-PDFF, MRE) | x | x | | | | | | x | | | x | | | x |
| Clinical laboratory tests (ALT, AST, GGT, ALP, albumin, butyrylcholinesterase, total bilirubin and platelets) | x | x | x | x | x | x | x | x | x | x | x | x | x | x |

Figure S2: Changes from baseline in serum albumin and butyrylcholinesterase in the MMRM sensitivity analysis.

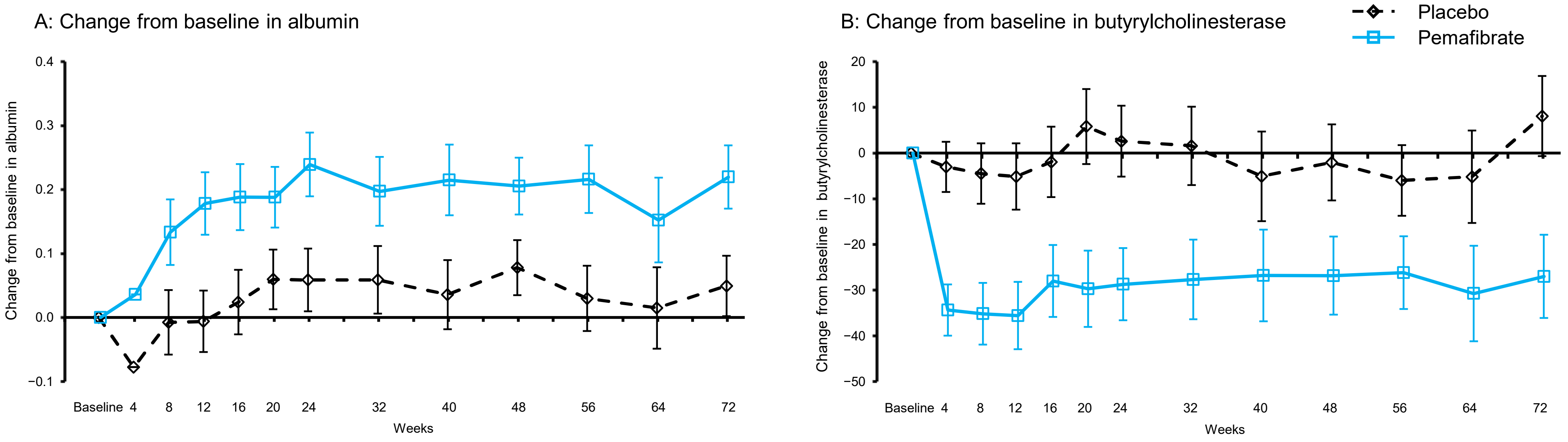


(A) Changes from baseline in serum albumin. (B) Changes from baseline in serum butyrylcholinesterase. Data are presented as least-squares means with 95% confidence intervals estimated using a mixed model for repeated measures (MMRM). MMRM, mixed model for repeated measures.

**Table S1:** Inclusion and exclusion criteria.

| | |
|---|---|
| Inclusion criteria | |
| (1) | Patients with the presence of liver fat content as defined by MRI-PDFF of ≥10% at Screening |
| (2) | Patients with the presence of liver stiffness as defined by MRE measurement of ≥2.5 kPa at Screening |
| (3) | Patients with the ALT level above the upper limit of normal (30 U/L for females and 40 U/L for males) at Screening |
| (4) | Patients with NAFLD aged 20 years or older when providing written informed consent (IC) |
| Exclusion criteria | |
| (1) | Current significant alcohol consumption defined as more than 210 g/week in males and more than 140 g/week in females on average |
| (2) | Planned use of contraindicated medications after providing written IC until the end of treatment |
| (3) | BMI <22 kg/m$^2$ at Screening |
| (4) | Uncontrolled diabetes mellitus as defined by HbA1c (NGSP) ≥8.0% at Screening |
| (5) | eGFR < 30 mL min$^{-1}$ 1.73$^{-1}$ m$^{-2}$ or on dialysis |
| (6) | Cirrhosis |
| (7) | Biliary obstruction |
| (8) | Evidence of the following other forms of liver disease:<br>•Hepatitis B or Hepatitis C<br>•Autoimmune hepatitis (AIH)<br>•Primary biliary cholangitis (PBC)<br>•Primary sclerosing cholangitis (PSC)<br>•Drug-induced liver injury<br>•Hyperthyroidism, Wilson's disease, hemochromatosis, alpha-1-antitrypsin disease |
| (9) | Patients with a malignant neoplasm or those judged by the investigator or subinvestigator to be at a high risk of recurrence |
| (10) | Patients with contraindications to MRI imaging |
| (11) | Patients who donated 200 mL or more of blood within 1 month before the administration of the study drug or 400 mL or more of blood within 4 months before the administration of the study drug |
| (12) | Patients with a history of serious drug allergies (such as anaphylactic shock) |
| (13) | Women with pregnancy, lactation, plan of pregnancy or women *1 of childbearing potential who do not use acceptable method of contraception *2 |
| (14) | Patients who were participating in another clinical study at the time of consent was obtained or who received study drugs other than the placebo less than 16 weeks before consent was obtained |
| (15) | Patients who have previously been administered Pemafibrate |
| (16) | Patients who have been determined inappropriate by the investigator or subinvestigator |

*1: Women of childbearing potential is defined as women who are biologically capable of pregnancy who have male partners that have not undergone contraceptive surgery. However, this excludes cases where the investigators have confirmed any of the following:

- The patient has undergone hysterectomy or tubal ligation prior to informed consent

- Women after menopause (women who have had no menstruation for at least 1 year for no other medical reasons)

*2: Acceptable method of contraception: Oral, implanted, or injected contraceptive hormones, mechanical devices (e.g., intrauterine device [IUD]), or a barrier method used in combination with spermicides (diaphragm, condom, cervical cap, etc.)

**Table S2:** Methodological details of serum butyrylcholinesterase measurements.

| Measured analyte | Central laboratory | Assay methodology | Analytical platforms | Unit | Reference range |
|---|---|---|---|---|---|
| Butyrylcholinesterase (pseudocholinesterase) | LSI Medience Corporation (Tokyo, Japan) | p-Hydroxybenzoylcholine method (JSCC-standardized method) | Automatic Analyzer LABOSPECT 008 $\alpha$ and BioMajesty JCA-BM8060 clinical chemistry analyzer | U/L | Male: 234–493 U/L<br>Female: 200–452 U/L |

**Table S3**: MMRM results for changes from baseline in serum albumin and butyrylcholinesterase.

| | Week 4 | Week 12 | Week 24 | Week 48 | Week 72 |
|---|---|---|---|---|---|
| Albumin (g/dL) | | | | | |
| Placebo | | | | | |
| Change | −0.03 [−0.08 to 0.02] | −0.01 [−0.05 to 0.04] | 0.06 [0.01 to 0.11] | 0.08 [0.03 to 0.12] | 0.05 [0.00 to 0.10] |
| *p* value | 0.274 | 0.801 | 0.019 | 0.001 | 0.041 |
| Pemafibrate | | | | | |
| Change | 0.09 [0.04 to 0.14] | 0.18 [0.13 to 0.23] | 0.24 [0.19 to 0.29] | 0.21 [0.16 to 0.25] | 0.22 [0.17 to 0.27] |
| *p* value | 0.001 | <0.001 | <0.001 | <0.001 | <0.001 |
| LS mean difference | | | | | |
| Estimate | 0.11 [0.04 to 0.19] | 0.19 [0.12 to 0.25] | 0.18 [0.11 to 0.25] | 0.13 [0.07 to 0.19] | 0.17 [0.10 to 0.24] |
| *p* value | 0.002 | <0.001 | <0.001 | <0.001 | <0.001 |
| Butyrylcholinesterase (U/L) | | | | | |
| Placebo | | | | | |
| Change | −3.0 [−8.5 to 2.5] | −5.1 [−12.4 to 2.1] | 2.6 [−5.2 to 10.4] | −2.0 [−10.4 to 6.3] | 8.1 [−0.7 to 16.9] |
| *p* value | 0.282 | 0.163 | 0.508 | 0.628 | 0.070 |
| Pemafibrate | | | | | |
| Change | −34.4 [−40.0 to −28.8] | −35.6 [−43.0 to −28.2] | −28.7 [−36.6 to −20.8] | −26.8 [−35.4 to −18.3] | −27.0 [−36.1 to −17.9] |
| *p* value | <0.001 | <0.001 | <0.001 | <0.001 | <0.001 |
| LS mean difference | | | | | |
| Estimate | −31.4 [−39.2 to −23.5] | −30.5 [−40.8 to −20.1] | −31.3 [−42.4 to −20.3] | −24.8 [−36.8 to −12.8] | −35.1 [−47.8 to −22.5] |
| *p* value | <0.001 | <0.001 | <0.001 | <0.001 | <0.001 |

Data are expressed as least-squares means (95% confidence intervals) for changes from baseline. Between-group differences are presented as least-squares mean differences (95% confidence intervals). The estimates were obtained using a mixed model for repeated measures (MMRM). No adjustment for multiplicity was performed; therefore, all *p* values should be interpreted as nominal. LS, least-squares; MMRM, mixed model for repeated measures.
